# Association of Serum Ustekinumab Levels With Clinical Response in Psoriasis

**DOI:** 10.1001/jamadermatol.2019.1783

**Published:** 2019-09-18

**Authors:** Teresa Tsakok, Nina Wilson, Nick Dand, Floris C. Loeff, Karien Bloem, David Baudry, Michael Duckworth, Shan Pan, Angela Pushpa-Rajah, Joseph F. Standing, Annick de Vries, Ali Alsharqi, Gabrielle Becher, Ruth Murphy, Shyamal Wahie, Andrew Wright, Christopher E. M. Griffiths, Nick J. Reynolds, Jonathan Barker, Richard B. Warren, A. David Burden, Theo Rispens, Deborah Stocken, Catherine Smith

**Affiliations:** 1 St John’s Institute of Dermatology, School of Basic & Medical Biosciences, Faculty of Life Sciences & Medicine, King’s College London, London, United Kingdom; 2 St John’s Institute of Dermatology, Guy’s and St Thomas’ National Health Service Foundation Trust, London, United Kingdom; 3 Institute of Health and Society, Faculty of Medical Sciences, Newcastle University, Newcastle-upon-Tyne, United Kingdom; 4 Department of Medical and Molecular Genetics, School of Basic & Medical Biosciences, Faculty of Life Sciences & Medicine, King’s College London, London, United Kingdom; 5 Department of Immunopathology, Sanquin Research and Landsteiner Laboratory, Amsterdam, the Netherlands; 6 Biologics Lab, Sanquin Diagnostic Services, Amsterdam, the Netherlands; 7 Infection, Immunity, Inflammation Section, University College London Great Ormond Street Institute of Child Health, London, United Kingdom; 8 Department of Dermatology, Royal Liverpool and Broadgreen University Hospital Trust, Liverpool, United Kingdom; 9 West Glasgow Ambulatory Care Hospital, Glasgow, United Kingdom; 10 Department of Dermatology, Queens Medical Centre, Nottingham University Teaching Hospitals, Nottingham, United Kingdom; 11 Department of Dermatology, University Hospital of North Durham, Durham, United Kingdom; 12 Centre for Skin Sciences, University of Bradford, Bradford, United Kingdom; 13 Dermatology Centre, Salford Royal National Health Service Foundation Trust, Manchester, United Kingdom; 14 The University of Manchester, Manchester Academic Health Science Centre, National Institute for Health Research Manchester Biomedical Research Centre, Manchester, United Kingdom; 15 Dermatology Sciences, Institute of Cellular Medicine, Medical School, Newcastle University, Newcastle upon Tyne, United Kingdom; 16 Department of Dermatology, Royal Victoria Infirmary, Newcastle Hospitals National Health Service Foundation Trust, Newcastle upon Tyne, United Kingdom; 17 Institute of Infection, Immunity and Inflammation, University of Glasgow, Glasgow, United Kingdom; 18 Leeds Institute of Clinical Trials Research, University of Leeds, Leeds, United Kingdom

## Abstract

**Importance:**

High-cost biologic therapies have transformed the management of immune-mediated inflammatory diseases. To optimize outcomes and reduce costs, dose adjustment informed by measurement of circulating drug levels has been shown to be effective in various settings. However, limited evidence exists for this approach with the interleukin 12 and interleukin 23 inhibitor ustekinumab.

**Objective:**

To evaluate clinical utility of therapeutic drug monitoring for ustekinumab in patients with psoriasis.

**Design, Setting, and Participants:**

A prospective observational cohort of 491 adults with psoriasis was recruited to the multicenter Biomarkers of Systemic Treatment Outcomes in Psoriasis study within the British Association of Dermatologists Biologic and Immunomodulators Register from June 2009 to December 2017; samples from some patients were taken between 2009 and 2011 as part of a pilot study with the same inclusion criteria.

**Exposure:**

Serum ustekinumab level measured at any point during the dosing cycle using an enzyme-linked immunosorbent assay.

**Main Outcomes and Measures:**

Disease activity measured using the Psoriasis Area and Severity Index (PASI) score. Treatment response outcomes were PASI75 (75% reduction in PASI score from baseline [primary outcome]), PASI90 (90% reduction of PASI score from baseline), and absolute PASI score of 1.5 or less.

**Results:**

A total of 491 patients (171 women and 320 men; mean [SD] age, 45.7 [12.8] years) had 1 or more serum samples (total, 853 samples obtained 0-56 weeks from start of treatment) and 1 or more PASI scores within the first year of treatment. Antidrug antibodies were detected in only 17 of 490 patients (3.5%). Early measured drug levels (1-12 weeks after starting treatment) were associated with PASI75 response 6 months after starting treatment (odds ratio, 1.38; 95% CI, 1.11-1.71) when adjusted for baseline PASI score, age, and ustekinumab dose. However, this finding was not consistent across the other PASI outcomes (PASI90 and PASI score of ≤1.5).

**Conclusions and Relevance:**

This real-world study provides evidence that measurement of early serum ustekinumab levels could be useful to direct the treatment strategy for psoriasis. Adequate drug exposure early in the treatment cycle may be particularly important in determining clinical outcome.

## Key Points

**Question:**

Can therapeutic drug monitoring for the interleukin-12 and interleukin-23 inhibitor ustekinumab optimize treatment pathways and outcomes in patients with psoriasis?

**Findings:**

This cohort study of 491 patients with psoriasis found that early serum ustekinumab levels were associated with a subsequent 75% reduction from baseline in Psoriasis Area and Severity Index score, although this association did not hold across other Psoriasis Area and Severity Index outcomes. Drug immunogenicity appeared to be low, with antidrug antibodies detected in only 17 of 490 patients (3.5%).

**Meaning:**

This study provides evidence that measurement of early ustekinumab levels could be useful to direct treatment strategy in patients with psoriasis; adequate drug exposure early in the treatment cycle may be particularly important in determining clinical outcome.

## Teaser

This cohort study evaluates clinical utility of monitoring serum levels of ustekinumab in patients with psoriasis.

## Introduction

Psoriasis is a chronic immune-mediated skin disease affecting at least 2% of the population.^1^ Management of psoriasis has been transformed by therapeutic monoclonal antibody biologics, of which the first-line choices are either adalimumab (a tumor necrosis factor inhibitor) or ustekinumab (an interleukin 12 [IL-12] and IL-23 inhibitor).^2^ There is wide variation in response to these drugs, with many patients not responding (primary treatment failure) or losing response over time (secondary treatment failure).^3,4^ Some of this heterogeneity may be explained by differences in the bioavailability and quantity of drug available at the target tissue, which in turn is influenced by adherence, drug dose, and pharmacokinetic covariates such as weight and drug immunogenicity (development of antidrug antibodies [ADAs]).

Unlike most other biologics used for inflammatory disease, ustekinumab is dosed according to body weight; patients who weigh less than 100 kg are generally given 45 mg of ustekinumab subcutaneously every 12 weeks, whereas those weighing at least 100 kg are given 90 mg subcutaneously every 12 weeks.^5^ Despite this dosing schedule, evidence suggests that ustekinumab dosing is suboptimal in some patients: clinical trial data previously showed that dose escalation increased rates of achieving 75% reduction from baseline in the Psoriasis Area and Severity Index (PASI) score (PASI75) in partial responders (those achieving ≥50% but <75% improvement from baseline PASI score),^6^ while patients with a higher baseline body mass index have been reported to receive in excess of the recommended cumulative dose during the first year of treatment.^7^ Similarly, response rates to ustekinumab in patients weighing 90 to 100 kg have been reported to be significantly lower than in other weight groups, suggesting that the standard 45-mg dose is inadequate in patients who are approaching the 100-kg threshold.^8^ On the other hand, ustekinumab dosing is likely to be excessive in some patients; a recent phase 3b study reported that lengthening intervals between ustekinumab doses did not affect maintenance of response.^9^ Taken together, these findings suggest that individualized dose optimization and therapeutic drug monitoring (TDM) of ustekinumab may have clinical utility.

Although several ustekinumab assays are commercially available in both the United States and Europe,^10,11,12,13,14,15^ monitoring of serum ustekinumab levels is not yet widely used in clinical practice. This is partly owing to limited evidence for TDM of this drug, in contrast to the strong correlation described between tumor necrosis factor inhibitor serum levels, ADAs, and treatment response across multiple immune-mediated inflammatory diseases.^16,17,18,19,20^ Reports on the association between ustekinumab level and response to treatment have been inconclusive,^21,22,23,24,25^ with basic parameter requirements for TDM (eg, therapeutic range and target drug level) yet to be established in the context of psoriasis.

Because the first step toward defining such parameters is to determine the association between drug levels and outcome, we investigated this using a real-world bioresource from the large multicenter cohort study BSTOP (Biomarkers of Systemic Treatment Outcomes in Psoriasis) within the UK pharmacovigilance registry BADBIR (British Association of Dermatologists Biologic and Immunomodulators Register). Specifically, we aimed to explore the association between drug level and response on the same day the drug level was measured, and to explore the association between early drug level and response at 6 months, because maximum clinical utility may lie in the ability to determine outcome and modify therapy prior to clinical relapse.

## Methods

### Patients and Setting

**Figure 1.**
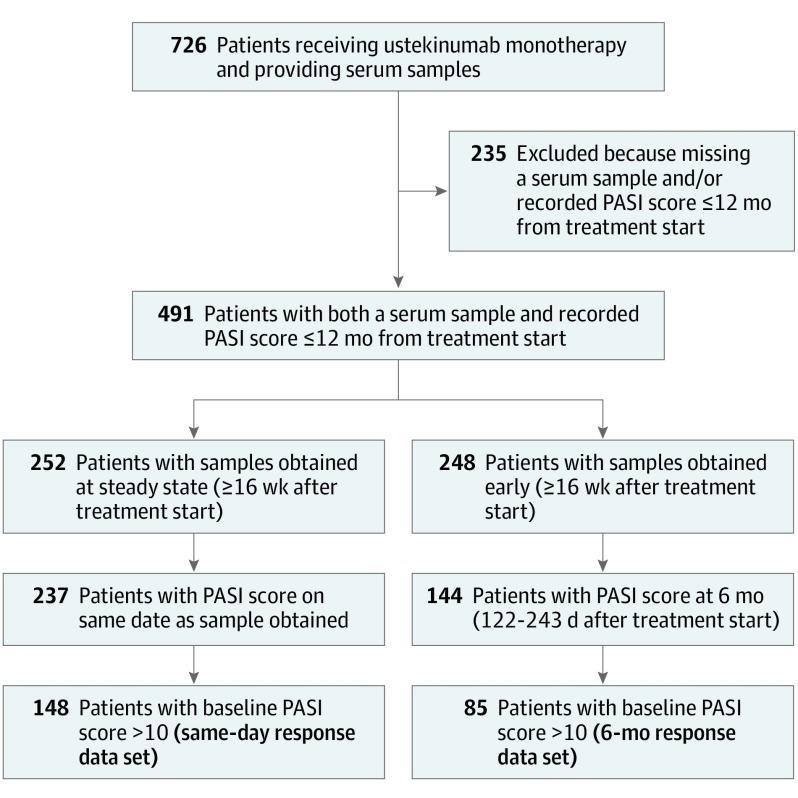
Flow Diagram of Patients Included in the Study PASI indicates Psoriasis Area and Severity Index.

As described previously,^20^ BSTOP is a prospective multicenter (n = 60) observational study, established in 2011 to identify markers of outcomes to systemic therapies for psoriasis. All UK adults fulfilling BSTOP inclusion criteria^26^ and enrolled in BADBIR^27^ were invited to participate. Venous blood samples were collected between June 2009 and December 2016 during routine clinic reviews; samples from some BSTOP patients were taken between 2009 and 2011 as part of a pilot study with the same inclusion criteria. Clinical response was assessed longitudinally using the PASI score. The current analysis includes patients receiving ustekinumab monotherapy, with 1 or more serum sample and 1 or more recorded PASI scores within the first year of treatment (Figure 1). This study was conducted in accordance with the 2008 Declaration of Helsinki.^28^ Three studies provided samples and data: a pilot study Predicting Drug Response (approved by National Research Ethics Service Committee London–South East 2; ethics approval code EC04/031), BSTOP (approved by National Research Ethics Service Committee London–South East 2; ethics approval code 11/H0802/7), and its nested study Psoriasis Stratification to Optimise Relevant Therapy Discovery (PSORTD) (approved by National Research Ethics Service Committee London–London Bridge; ethics approval code 14/LO/1685). Written informed consent was obtained from all participants before enrollment.

### Drug Level and ADA Measurements

Venous blood was collected during clinic reviews and centrifuged for 10 minutes (2000*g*) and serum aliquots were frozen (–80°C). In this pragmatic study, samples were not collected from every patient at every time point; most were collected without reference to treatment administration. Samples within the first year of treatment were sent to Sanquin for measurement of ustekinumab levels and ADAs. The ustekinumab level assay was an enzyme-linked immunosorbent assay similar to a previously developed adalimumab assay,^29^ but using IL-12 to capture ustekinumab, with rabbit anti-ustekinumab for detection (lower limit of detection, 0.02 μg/mL). Antidrug antibodies were measured using a previously described radioimmunoassay,^30^ with minor modifications (ADA positive cutoff >12 arbitrary units/mL). Specifically, 1 μL of serum diluted in freeze medium was incubated with Sepharose-immobilized protein A in the presence of 1-ng/test biotin-conjugated ustekinumab F(ab)_2_. Nonbound serum components were removed by washing before 50 μL of iodine 125–labeled streptavidin was added in a 500-μL phosphate-buffered saline-albumin solution (0.3% bovine serum albumin, 0.01M EDTA, 0.004% polysorbate 20, and 0.05% sodium azide). After incubation and washing, radioactivity was measured using a gamma counter. Assay results were converted to arbitrary units/mL calculated from a 2-fold serially diluted calibration curve of a polyclonal ustekinumab-specific rabbit anti-idiotype.^31^ This assay format has limited drug tolerance^32^ but was previously shown to have better correlation with clinical response vs drug-tolerant alternatives in patients with rheumatoid arthritis who were taking adalimumab.^33^

### Outcome Measures

Primary treatment response was defined as achieving PASI75, with baseline PASI score defined as the most recent PASI score recorded prior to the date of the first drug dose within the preceding 6 months.^3,34^ Secondary outcomes were 90% reduction in PASI score from baseline (PASI90) and PASI score of 1.5 or less (absolute PASI score of ≤1.5, which approximates to PASI90; written communication, Nina Wilson, PhD, January 2019).

### Statistical Analysis

Based on previous work using adalimumab drug levels,^20^ we explored the association between ustekinumab level and response in 2 ways. First, we investigated the association between drug levels and response on the same day of the sample; second, we investigated whether drug levels sampled early after treatment start are associated with response at 6 months. Two data sets were therefore derived: a data set comprising samples obtained at steady state (≥16 weeks after treatment start), with a corresponding PASI score on the same day as the sample date, hereafter referred to as the same-day response data set, and a data set comprising samples obtained early in the treatment course (1-12 weeks after treatment start), with a corresponding PASI score at 6 months (122-243 days after treatment start), hereafter referred to as the 6-month response data set. Analyses for PASI75 and PASI90 responses were restricted to patients with a baseline PASI score higher than 10 as an accepted criterion for severe disease,^35^ and to minimize confounding due to prebiologic treatments. The latter is particularly relevant in this real-world data set.

#### Descriptive Analysis

A descriptive concentration effect curve was generated to assess whether clinical response plateaus beyond a certain drug level. Box plots were used to visually compare drug levels by responder group in both the same-day response and 6-month response data sets.

#### Logistic Regression Analysis

We used univariate logistic regression models with the 6-month response data set to explore the association between early drug levels and treatment response in the presence of other covariates, including those previously identified as factors associated with response in the BADBIR cohort (eg, weight, race/ethnicity, disease and treatment duration, ustekinumab dose, and biologic-naïve status).^36^ Given that most samples were not trough levels, we also included time of sample from last ustekinumab dose as a covariate. For continuous covariates, the best-fitting simple nonlinear transformation was chosen based on reduction in the Akaike Information Criterion. Covariates associated with response at significance level of *P* < .10 were taken forward to a multivariable logistic regression model. Forward selection techniques were then used, with covariate inclusion based on a significance level of *P* < .05. Multivariable models were derived for all 3 PASI outcomes (PASI75, PASI90, and PASI score of ≤1.5). For PASI90 and PASI score of 1.5 or less, drug level was included as the first covariate and retained at each stage, despite not being significant on univariate analysis. Pseudo *R*^2^ and Akaike Information Criterion were calculated to assess model fit. All analyses were undertaken using Stata, version 14,^37^ on a complete case basis.

## Results

### Patient Cohort and Baseline Characteristics

A total of 491 patients receiving ustekinumab monotherapy had both serum samples and PASI scores available within the first year of treatment (Figure 1, Table 1). The cohort was predominantly male (320 [65.2%]), with a mean (SD) body mass index (calculated as weight in kilograms divided by height in meters squared) of 32.0 (7.3) and mean (SD) baseline PASI score of 13.3 (6.8). A total of 201 patients (40.9%) were biologic-naive, and 282 (57.4%) were receiving 45 mg of ustekinumab vs 209 (42.6%) receiving 90 mg (Table 1). Patients not providing serum samples were excluded, but their baseline characteristics were similar (eTable 1 in the Supplement).

**Table 1.** Summary Statistics for the Full Cohort, Same-Day Response Data Set, and 6-Month Response Data Set

Covariate	Full Cohort (491 Patients; 853 Samples)		Response Data Set			
			Same Day (148 Patients; 175 Samples)^a^		At 6 mo (85 Patients; 119 Samples)^a^	
	Mean (SD)	Complete Data, No. (%)	Mean (SD)	Complete Data, No. (%)	Mean (SD)	Complete Data, No. (%)
Baseline PASI score	13.3 (6.8)	452 (92.1)	16.6 (5.2)	148 (100)	16.3 (5.5)	85 (100)
Height, cm	172.2 (10.3)	463 (94.3)	172.4 (10.5)	140 (94.6)	172.1 (10.5)	81 (95.3)
Weight, kg	94.7 (22.7)	435 (88.6)	96.1 (23.7)	140 (94.6)	94.2 (22.9)	80 (94.1)
Waist, cm	105.8 (16.8)	420 (85.5)	106.5 (17.4)	131 (88.5)	105.2 (15.7)	77 (90.6)
BMI	32.0 (7.3)	427 (87.0)	32.3 (7.7)	136 (91.9)	31.7 (7.6)	78 (91.8)
Age, y	45.7 (12.8)	491 (100)	45.2 (13.1)	148 (100)	48.7 (13.3)	85 (100)
Disease duration, y	23.3 (13.1)	464 (94.5)	23.1 (13.1)	142 (95.9)	23.4 (13.0)	82 (96.5)
	**No. (%)**		**No. (%)**		**No. (%)**	
White race/ethnicity	421 (85.7)	491 (100)	123 (83.1)	148 (100)	70 (82.4)	85 (100)
						
Male sex	320 (65.2)	491 (100)	99 (66.9)	148 (100)	59 (69.4)	85 (100)
Inflammatory arthritis	101 (23.5)	430 (87.6)	26 (18.8)	138 (93.2)	24 (30.4)	79 (92.9)
Ever smoked	289 (61.2)	472 (96.1)	81 (55.9)	145 (98.0)	51 (61.4)	83 (97.6)
Palm psoriasis	93 (21.1)	441 (89.8)	30 (21.6)	139 (93.9)	19 (24.1)	79 (92.9)
Biologic naive	201 (40.9)	491 (100)	64 (43.2)	148 (100)	37 (43.5)	85 (100)
Dose		491 (100)		148 (100)		85 (100)
45 mg	282 (57.4)	NA	82 (55.4)	NA	48 (56.5)	NA
90 mg	209 (42.6)	NA	66 (44.6)	NA	37 (43.5)	NA

Abbreviations: BMI, body mass index (calculated as weight in kilograms divided by height in meters squared); NA, not applicable; PASI, Psoriasis Area and Severity Index.

^a^ Summaries for the same-day response and 6-month response data sets are restricted to patients with a baseline PASI score higher than 10.

### Response to Treatment

A total of 348 patients (70.9%) achieved PASI75 at some point within a year of starting treatment. PASI75 remains a standard measure of adequate treatment response in UK guidelines.^38^

### Drug Levels and ADAs

Drug levels were sampled according to standard clinical care. Excluding samples obtained on the day the first dose was given, the median time from last dose was 28 days (interquartile range [IQR], 16-57 days; range, 0-98 days; data available for 515 samples), median drug level was 1.19 μg/mL (IQR, 0.37-2.86 μg/mL; range 0-13.1 μg/mL; 800 samples), and ADAs were detected in 17 of 490 patients (3.5%) in 20 samples obtained 29 to 350 days after starting treatment.

### Relationship Between Drug Level and Response

All analyses considered all eligible samples. There was a maximum of 4 samples per patient.

#### Descriptive Analysis

**Figure 2.**
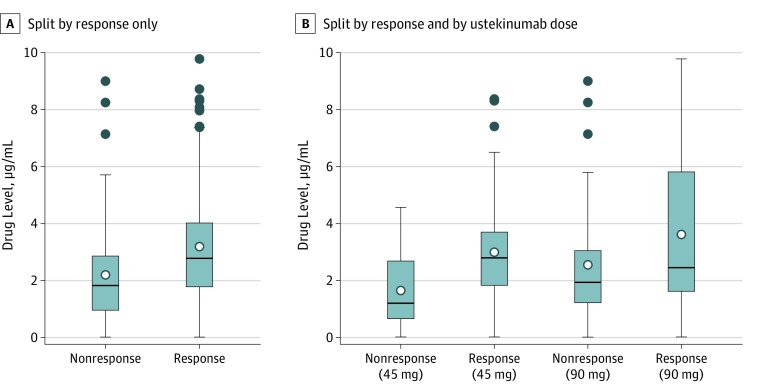
Box Plots Comparing Early Measured Drug Levels by Achievement of 75% Reduction From Baseline in Psoriasis Area and Severity Index at 6 Months A, Split by response only. The nonresponse group contains 46 samples and the response group contains 73 samples. B, Split by response and by ustekinumab dose. The nonresponse group receiving 45 mg of ustekinumab contains 18 samples, the response group receiving 45 mg of ustekinumab contains 50 samples, the nonresponse group receiving 90 mg of ustekinumab contains 28 samples, and the response group receiving 90 mg of ustekinumab contains 23 samples. In both panels, the middle line is the median, white circles are the means, ends of boxes are the lower and upper quartiles, dark blue circles are outliers (values ≥1.5 times the interquartile range from the lower and upper quartiles), and whiskers show the minimum and maximum values (unless there are outliers, in which case they are 1.5 times the interquartile range from the lower and upper quartiles).

A concentration effect curve showed no clear evidence of an association between steady state drug levels and same-day absolute PASI (eFigure 1 in the Supplement). Median drug level and spread of drug levels were similar between patients recorded to have responded and those who did not respond on the same day as the serum sample was obtained (same-day response data set; eFigure 2 in the Supplement). However, patients achieving PASI75 at 6 months (6-month response data set) on average had higher early ustekinumab levels (median, 2.78 μg/mL; IQR, 1.78-4.02 μg/mL; range, 0.02-9.78 μg/mL) compared with patients not achieving PASI75 (median, 1.83 μg/mL; IQR, 0.96-2.86 μg/mL; range, 0.02-9.00 μg/mL) (Figure 2A), with overlapping ranges between the 2 groups. A similar pattern was observed for the other 2 response outcomes, PASI90 and PASI score of 1.5 or less (eFigure 3 in the Supplement).

To explore the association between drug level, response, and dose, we split box plot data by ustekinumab doses of 45 mg and 90 mg (Figure 2B). As expected, patients who achieved PASI75 had higher median drug levels than did nonresponders within each dose group. This pattern was also evident for the outcomes of PASI90 and PASI score of 1.5 or less (eFigure 4 in the Supplement). However, patients who did not achieve PASI75 while taking 90 mg of ustekinumab had slightly higher median drug levels than did nonresponders taking 45 mg of ustekinumab, albeit with overlapping ranges and large variability (Figure 2B).

#### Logistic Regression Analysis

**Figure 3.**
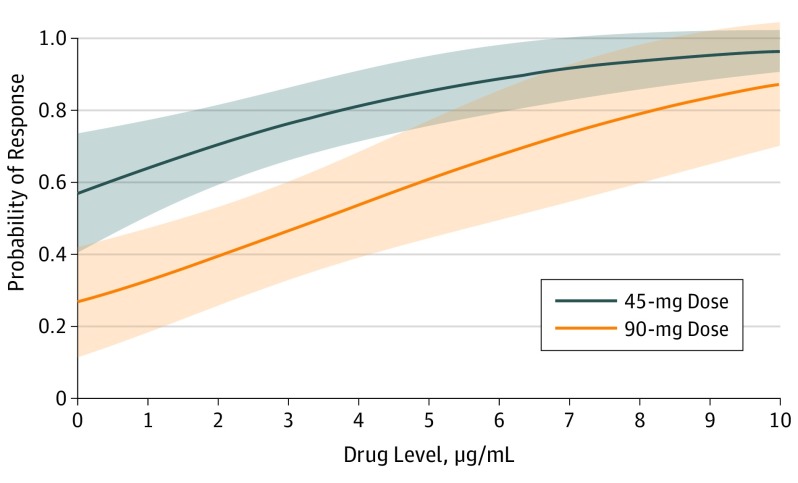
Probability of Achieving 75% Reduction From Baseline in Psoriasis Area and Severity Index Score at 6 Months Based on Early Measured Drug Level, Split by Ustekinumab Dose Probability of response is split by ustekinumab dose. Solid lines plot the marginal predicted probability of response; the shaded areas indicate 95% CIs.

Univariate logistic regression indicated that early drug level was associated with 6-month PASI75 (6-month response data set: odds ratio, 1.27; 95% CI, 1.04-1.56), but there was no evidence of this association for the other 2 PASI outcomes (eTable 2 in the Supplement). Next, multivariable models were derived to explore the association between early drug level and 6-month response in the presence of other relevant covariates. The final model for PASI75 included drug dose, baseline PASI score, and age as well as drug level (odds ratio, 1.38; 95% CI, 1.11-1.71) (Table 2), and shows increasing probability of response with increasing drug level (Figure 3). The model also suggests that patients taking the higher ustekinumab dose (90 mg) have a lower probability of response for a given drug level (Figure 3). To explore this finding further, we inspected box plots of drug levels split by weight and dose (eFigure 5 in the Supplement). Despite overlapping ranges, these box plots show slightly lower median drug levels in patients weighing more than 100 kg and in patients taking the higher ustekinumab dose.

**Table 2.** Final Multivariable Models for Determining 6-Month Response

Covariate	Coefficient (SE)	OR (95% CI)	*P* Value	Pseudo-*R*^2^	Samples, No.	Responders, No. (% of Samples)
PASI75						
Drug level	0.32 (0.11)	1.38 (1.11-1.71)	.004	0.18	119	73 (61.3)
Baseline PASI score	0.10 (0.04)	1.10 (1.01-1.20)	.03			
Age	0.04 (0.02)	1.04 (1.00-1.07)	.03			
90-mg Dose	−1.43 (0.44)	0.24 (0.10-0.56)	.001			
PASI90						
Drug level	0.14 (0.09)	1.15 (0.97-1.38)	.11	0.10	115	45 (39.1)
Baseline PASI score	0.10 (0.04)	1.11 (1.02-1.20)	.01			
Disease duration	0.04 (0.02)	1.04 (1.01-1.08)	.009			
PASI score ≤1.5						
Drug level	0.11 (0.08)	1.12 (0.96-1.30)	.15	0.06	186	58 (31.2)
Naive to biologics	0.92 (0.33)	2.51 (1.31-4.81)	.006			
Ever smoked	−0.70 (0.34)	0.50 (0.26-0.96)	.04			

Abbreviations: OR, odds ratio; PASI75, 75% reduction from baseline in Psoriasis Area and Severity Index score; PASI90, 90% reduction from baseline in Psoriasis Area and Severity Index score.

Drug level was nonsignificant for the outcomes of PASI90 and PASI score of 1.5 or less, even taking into account other covariates. Furthermore, significant covariates were not consistent across the 3 models for different PASI outcomes within the 6-month response data set. Finally, we performed a sensitivity analysis by fitting the final model for PASI75 to very early trough samples (21-28 days after treatment start). Despite smaller sample size and greater uncertainty around estimates, a similar association was seen between drug level and response (odds ratio, 3.71; 95% CI, 1.24-11.08) (eTable 3 in the Supplement).

## Discussion

### Key Findings

To our knowledge, this is the largest study to date of ustekinumab drug level monitoring in patients with psoriasis. We report evidence that early ustekinumab levels were significantly associated with 6-month PASI75 response. This finding has particular clinical and practical relevance because assays to measure serum ustekinumab levels are already commercially available in both the United States and Europe.^10,11,12,13,14,15^

We also report a low rate (3.5%) of detectable ADAs to ustekinumab within the first year of treatment, compared with the previously reported rate of 37.5% in a cohort of patients taking adalimumab that was derived from the same UK study.^20^ It is possible that this differential drug immunogenicity accounts, at least partially, for significantly higher rates of drug survival (length of time from initiation to discontinuation of treatment) in patients taking ustekinumab compared with those taking adalimumab.^3^

The finding that the higher ustekinumab dose is associated with a lower probability of response is perhaps surprising. One possible explanation is that patients taking the higher dose exhibit characteristics associated with poor response that have not been accounted for in our model. An alternative explanation may be that a double dose of ustekinumab (90 mg vs 45 mg) fails to adequately compensate for the increased volume of distribution in some people with a higher body weight; we noted that median drug levels were slightly lower in patients taking the higher ustekinumab dose and in patients weighing more than 100 kg.

Our data set should allow for stable estimation of comparable numbers (4-5) of covariates^39^ in each of the analyses for early drug level vs the 3 different PASI outcomes. However, we were unable to demonstrate a link between early drug level and the other PASI outcomes, nor between steady state drug levels and same-day response. It is therefore possible that the association between early drug level and PASI75 is owing to a spurious *P* value or statistical artifact. This contrasts with findings for adalimumab,^14^ where the same statistical approach showed that both early and steady state drug levels were associated with all PASI outcomes. A fundamental explanation for this may lie in differing mechanisms of biologic action: adalimumab directly inhibits the inflammatory effector cytokine tumor necrosis factor, whereas ustekinumab inhibits IL-12 and IL-23, with the latter being a master regulator of pathogenic T helper 17 cell development.^40^ Just as the underlying biological effect is more complex for ustekinumab, it may be that the association between drug level and response is correspondingly convoluted.

### Existing Literature

To our knowledge, there are few other studies in this area, generally limited to descriptive or empirical analyses investigating the association between ustekinumab level and response, which report mixed results. The most recent study in psoriasis included prospective follow-up of only 27 patients, but reported similar findings to ours in that very early drug levels (week 6) were inversely correlated with subsequent response (week 12).^25^ However, in line with our data, no association was detected between drug levels measured later (in this case, at week 12) and same-day response.

The largest study in patients with psoriasis reported significantly lower drug levels and PASI50 response rates in patients with detectable ADAs compared with those without detectable ADAs.^23^ Finally, in a Dutch cohort of 41 patients with psoriasis, there was no correlation between ustekinumab level and response; 3 of 41 patients (7.3%) developed ADAs.^24^

Larger-scale studies have been conducted in the context of inflammatory bowel disease. It is possible that variability in the amount of drug lost via the inflamed gut means that some patients are less able to achieve adequate serum concentrations, meaning that TDM may have greater utility in this setting. An analysis of phase 3 trial data (n = 1154) reported a positive association of drug levels with clinical and endoscopic improvement, and an inverse correlation with C-reactive protein level.^21^ Only 2% of patients developed ADAs.

### Strengths and Limitations

A strength of this study is high external validity, as more than 50% of all UK patients with psoriasis taking biologics are registered in BADBIR, and 95% of UK dermatology centers prescribing biologics for psoriasis contribute data to BADBIR. Our findings highlight the potential clinical utility of this easily measurable early biomarker in optimizing subsequent response. They also serve as a call to action for both industry and academia to develop cost-effective and widely available assays, and to further validate the role of TDM in clinical practice.

One limitation of our study is that, of 491 patients with both a serum sample and PASI score within 1 year of treatment, the same-day response data set included 148 patients and the 6-month response data set included 85 patients. Figure 1 shows the dropoff in patient numbers at each stage of filtering.

A second limitation relates to the difficulty in accounting for the complex association between drug level and response using standard logistic regression modeling. This approach has been successfully used in other settings, notably to define a therapeutic range and target drug level for adalimumab.^20^ However, it is possible that ustekinumab’s extended dosing interval compared with adalimumab may pose a particular hindrance in this context, as a single or small number of drug levels may represent a relatively poor measure of total drug exposure. This issue may have been exacerbated by pragmatic serum sampling and PASI assessment at routine clinical visits, as opposed to having samples measured and PASI assessments performed only during trough periods. To partially address this issue, we accounted for the timing of samples by including time from last ustekinumab dose as a covariate in modeling, but this did not remain in the final multivariable models after the forward selection process. Finally, the validity of our findings is limited to within 1 year of the start of treatment, as this was the selected cohort duration.

## Conclusions

Despite the complexities outlined above, we did find a statistically significant association between early drug levels (≤12 weeks) and 6-month PASI75 response in patients with psoriasis taking ustekinumab. This finding suggests that adequate drug exposure early in the treatment cycle may be particularly important in determining clinical outcome with ustekinumab. However, our statistical approach did not take into account patient-level pharmacokinetic parameters such as volume of distribution and clearance, nor potential differences in evolution of PASI score over time vs changing drug levels. Therefore, future work should focus on pharmacokinetic-pharmacodynamic modeling of the whole time course of response to ustekinumab.^41^ This modeling may be of particular relevance for biologics with more upstream targets, such as differentiation pathway cytokines as opposed to effector cytokines. Further investigation to confirm the clinical utility of TDM of ustekinumab and other biologics is a key step toward personalization of treatment regimens across multiple immune-mediated inflammatory diseases.

## Notes

Supplement. **eTable 1.** Summary Statistics for Patients not Providing Serum Samples During the First 12 Months on Ustekinumab **eTable 2.** Univariate Analyses for Predicting 6-Month Response Based on Early Drug Level **eTable 3.** Very Early (4-Week) Drug Levels Predicting PASI75 Response at 6 Months **eFigure 1.** Concentration Effect Curve of Median Percentage Change in PASI Against Median Drug Level (Same-Day Response Dataset) **eFigure 2.** Boxplots Comparing Drug Levels by Same-Day Response **eFigure 3.** Boxplots Comparing Early Drug Levels by 6-Month Response **eFigure 4.** Boxplots Comparing Early Drug Levels by 6-Month Response, Split by Ustekinumab Dose **eFigure 5.** Boxplots Comparing Early Drug Levels by Weight and Ustekinumab Dose
