# Supplementary material for: Association of Serum Ustekinumab Levels With Clinical Response in Psoriasis
Source: JAMA Dermatol. 2019 Sep 18;155(11):1235–43. doi: 10.1001/jamadermatol.2019.1783 (PMC6751771; doi:10.1001/jamadermatol.2019.1783)
Supplement: Supplement. — eTable 1. Summary Statistics for Patients not Providing Serum Samples During the First 12 Months on Ustekinumab eTable 2. Univariate Analyses for Predicting 6-Month Response Based on Early Drug Level eTable 3. Very Early (4-Week) Drug Levels Predicting PASI75 Response at 6 Months eFigure 1. Concentration Effect Curve of Median Percentage Change in PASI Against Median Drug Level (Same-Day Response Dataset) eFigure 2. Boxplots Comparing Drug Levels by Same-Day Response eFigure 3. Boxplots Comparing Early Drug Levels by 6-Month Response eFigure 4. Boxplots Comparing Early Drug Levels by 6-Month Response, Split by Ustekinumab Dose eFigure 5. Boxplots Comparing Early Drug Levels by Weight and Ustekinumab Dose [file jamadermatol-155-1235-s001.pdf]

Supplementary Online Content

Tsakok T, Wilson N, Dand N, et al; British Association of Dermatologists Biologic and Immunomodulators Register (BADBIR) Study Group; Psoriasis Stratification to Optimise Relevant Therapy (PSORT) Consortium. Association of serum ustekinumab levels with clinical response in psoriasis. *JAMA Dermatol.* Published online September 18, 2019. doi:10.1001/jamadermatol.2019.1783

eTable 1. Summary Statistics for Patients not Providing Serum Samples During the First 12 Months on Ustekinumab
eTable 2. Univariate Analyses for Predicting 6-Month Response Based on Early Drug Level
eTable 3. Very Early (4-Week) Drug Levels Predicting PASI75 Response at 6 Months
eFigure 1. Concentration Effect Curve of Median Percentage Change in PASI Against Median Drug Level (Same-Day Response Dataset)
eFigure 2. Boxplots Comparing Drug Levels by Same-Day Response
eFigure 3. Boxplots Comparing Early Drug Levels by 6-Month Response
eFigure 4. Boxplots Comparing Early Drug Levels by 6-Month Response, Split by Ustekinumab Dose
eFigure 5. Boxplots Comparing Early Drug Levels by Weight and Ustekinumab Dose

This supplementary material has been provided by the authors to give readers additional information about their work.

© 2019 Tsakok T et al. *JAMA Dermatol*.

eTable 1. Summary Statistics for Patients not Providing Serum Samples During the First 12 Months on Ustekinumab

	**Patients not providing serum samples** (n= 309 patients)	
**Covariate**	**Mean (sd)**	**Complete data n (%)**
Baseline PASI	13.5 (8.5)	268 (86.7)
Height (cm)	169.7 (11.4)	281 (90.9)
Weight (kg)	92.5 (21.8)	280 (90.6)
Waist (cm)	102.8 (16.6)	257 (83.2)
BMI (kg/m^2)	32.1 (7.4)	265 (85.8)
Age (years)	44.4 (12.2)	309 (100.0)
Disease duration (years)	23.4 (12.2)	283 (91.6)
	**n (%)**	
Ethnicity – white	272 (88.0)	309 (100.0)
Gender – male	179 (57.9)	309 (100.0)
Inflammatory arthritis	76 (26.3)	289 (93.5)
Ever smoked	192 (64.4)	298 (96.4)
Palm psoriasis	56 (19.4)	289 (93.5)
Biologic naive	96 (31.1)	309 (100.0)
Dose 45mg<br>90mg	182 (60.1)<br>121 (39.9)	303 (98.1)

© 2019 Tsakok T et al. *JAMA Dermatol*.

eTable 2. Univariate Analyses for Predicting 6-Month Response Based on Early Drug Level

	PASI75			PASI90			PASI≤1.5		
**Covariate**	**Coef (s.e)**	**OR (95% CI)**	**P value**	**Coef (s.e)**	**OR (95% CI)**	**P value**	**Coef (s.e)**	**OR (95% CI)**	**P value**
Drug level (µg/ml)	0.24 (0.10)	1.27 (1.04,1.56)	0.02	0.14 (0.09)	1.15 (0.97,1.36)	0.11	0.13 (0.07)	1.14 (0.99,1.32)	0.07
Time of sample from last dose (days)	-0.02 (0.01)	0.98 (0.96,1.00)	0.05	-0.02 (0.01)	0.98 (0.96,1.00)	0.03	-0.02 (0.01)	0.98 (0.96,1.00)	0.01
Dose 90 mg	-1.22 (0.39)	0.30 (0.14,0.64)	0.002	-0.55 (0.39)	0.58 (0.27,1.24)	0.16	-0.50 (0.33)	0.61 (0.32,1.16)	0.13
Baseline PASI	0.08 (0.04)	1.08 (1.00,1.16)	0.05	0.07 (0.03)	1.07 (1.00,1.15)	0.04	0.03 (0.02)	1.03 (0.98,1.08)	0.2
Disease duration (years)	0.02 (0.02)	1.02 (0.99,1.06)	0.11	0.04 (0.02)	1.04 (1.01,1.07)	0.02	0.02 (0.01)	1.02 (0.99,1.04)	0.14
Treatment duration (days)	-0.008 (0.008)	0.99 (0.98,1.01)	0.3	-0.009 (0.008)	0.99 (0.98,1.01)	0.3	-0.008 (0.007)	0.99 (0.98,1.01)	0.2
Biologic naive	0.31 (0.38)	1.37 (0.65,2.86)	0.4	0.26 (0.38)	1.29 (0.62, 2.71)	0.5	0.93 (0.32)	2.54 (1.35,4.76)	0.004
Age (years)	0.03 (0.01)	1.03 (1.00, 1.05)	0.07	0.002 (0.014)	1.00 (0.98,1.03)	0.9	-0.002 (0.012)	1.00 (0.98,1.02)	0.9
Gender – male	-0.68 (0.42)	0.51 (0.22,1.15)	0.11	-0.31 (0.40)	0.74 (0.34,1.61)	0.4	-0.13 (0.33)	0.88 (0.46,1.69)	0.7
Ethnicity – white	-0.27 (0.54)	0.76 (0.26,2.20)	0.6	-0.81 (0.52)	0.44 (0.16,1.22)	0.12	0.77 (0.48)	2.17 (0.84,5.59)	0.11
Height (cm)	-0.03 (0.02)	0.97 (0.93,1.00)	0.08	-0.03 (0.02)	0.97 (0.93,1.00)	0.09	-0.03 (0.02)	0.97 (0.94,1.01)	0.10
Weight (kg)	-0.004 (0.008)	1.00 (0.98,1.01)	0.7	0.002 (0.008)	1.00 (0.99, 1.02)	0.8	-0.003 (0.007)	1.00 (0.98,1.01)	0.7
Weight ≥100kg	-0.11 (0.41)	0.89 (0.40,1.99)	0.8	-0.34 (0.41)	0.72 (0.32,1.61)	0.4	-0.16 (0.35)	0.85 (0.43,1.69)	0.6
Waist (cm)	-0.003 (0.012)	1.00 (0.97,1.02)	0.8	0.002 (0.012)	1.00 (0.98,1.03)	0.8	-0.02 (0.01)	0.98 (0.96,1.00)	0.12
BMI (kg/m^2)	0.01 (0.03)	1.01 (0.97,1.07)	0.6	0.03 (0.02)	1.03 (0.98,1.08)	0.18	0.01 (0.02)	1.01 (0.97,1.06)	0.6
Inflammatory arthritis	-0.25 (0.42)	0.78 (0.34,1.76)	0.5	-0.80 (0.45)	0.45 (0.19,1.09)	0.08	-0.45 (0.38)	0.64 (0.30,1.35)	0.2

© 2019 Tsakok T et al. *JAMA Dermatol*.

Palm psoriasis	0.51 (0.51)	1.66 (0.61,4.52)	0.3	0.48 (0.49)	1.61 (0.62,4.22)	0.3	-0.16 (0.41)	0.85 (0.38,1.91)	0.7
Ever smoked	-0.30 (0.40)	0.74 (0.34,1.62)	0.5	-0.69 (0.39)	0.50 (0.23,1.09)	0.08	-0.58 (0.32)	0.56 (0.30,1.05)	0.07
Smoking at baseline	0.44 (0.46)	1.56 (0.63,3.82)	0.3	-0.18 (0.44)	0.83 (0.35,1.97)	0.7	-0.66 (0.41)	0.52 (0.23,1.14)	0.10
Alcohol at baseline	0.62 (0.47)	1.86 (0.75,4.66)	0.18	0.51 (0.49)	1.66 (0.64, 4.31)	0.3	0.62 (0.45)	1.85 (0.77,4.47)	0.17

For PASI75 and PASI90, there are 119 samples on 85 patients after restricting to baseline PASI>10. For PASI≤1.5, there are 191 samples on 144 patients, since baseline PASI is not needed to calculate response. Accordingly, for this PASI outcome we did not restrict to patients with baseline PASI>10.

© 2019 Tsakok T et al. *JAMA Dermatol*.

eTable 3. Very Early (4-Week) Drug Levels Predicting PASI75 Response at 6 Months

The model from Table 2 has been fitted to samples between 21-28 days. There are 39 samples on 39 patients.

	Covariate	Coefficient (s.e)	OR (95% CI)	P value	Pseudo R^2	Number of samples	Number of responders (% of samples)
**PASI75**	Drug level (µg/ml)	1.31 (0.56)	3.71 (1.24,11.08)	0.02	0.43	39	27 (69.2)
	Baseline PASI	0.41 (0.19)	1.51 (1.03,2.21)	0.03			
	Age (years)	0.05 (0.04)	1.05 (0.97,1.15)	0.2			
	Dose 90 mg	-2.26 (1.13)	0.10 (0.01,0.96)	0.05			

© 2019 Tsakok T et al. *JAMA Dermatol*.

eFigure 1. Concentration Effect Curve of Median Percentage Change in PASI Against Median Drug Level (Same-Day Response Dataset)

These summaries are calculated for approximately equally sized groups of observations with similar drug levels (apart from the first group, which includes all drug levels below the limit of detection, 0.02 µg/ml). Note that the maximum drug level in the highest group is 7.54 µg/ml. Vertical bars: interquartile range (IQR); grey horizontal lines: PASI75 and PASI90 response.

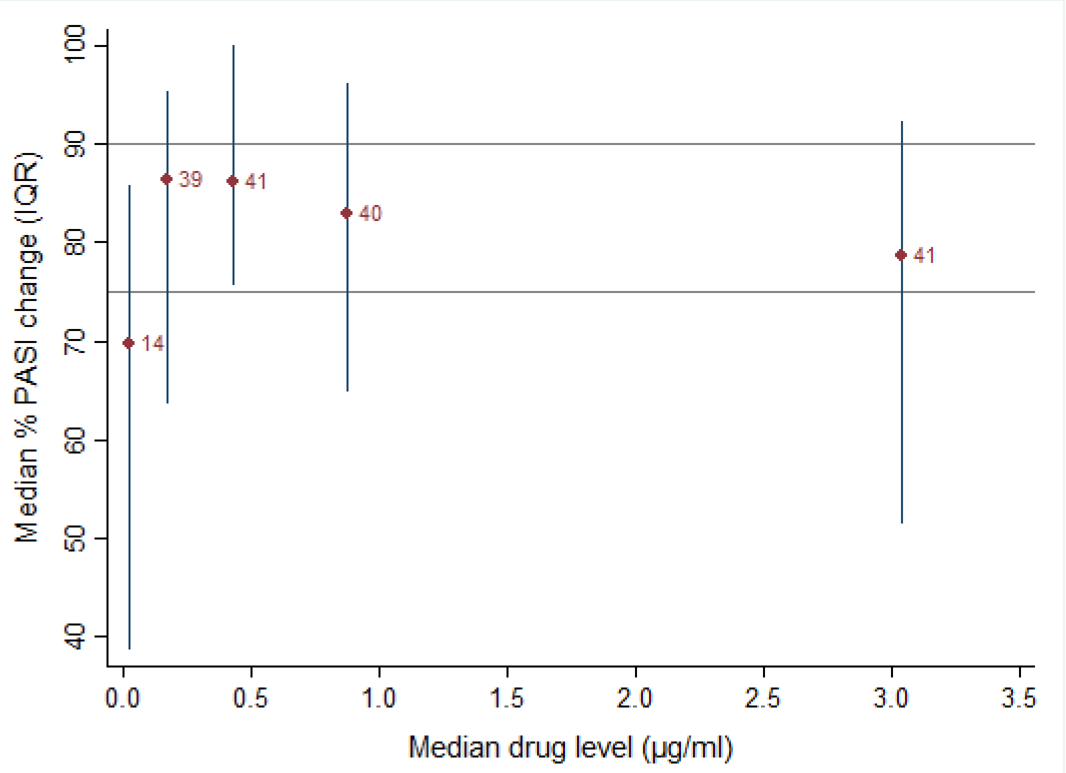

© 2019 Tsakok T et al. *JAMA Dermatol*.

eFigure 2. Boxplots Comparing Drug Levels by Same-Day Response

(a) PASI75 response vs non-response

n = 69 and 106 samples in each group respectively

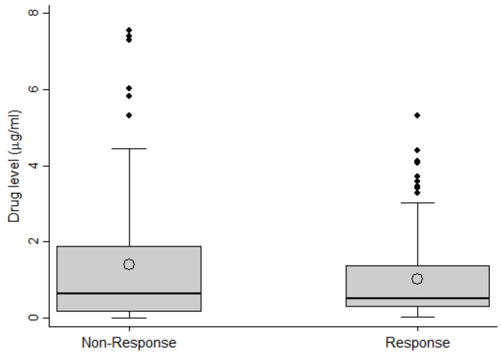

(b) PASI90 response vs non-response

n = 120 and 55 samples in each group respectively

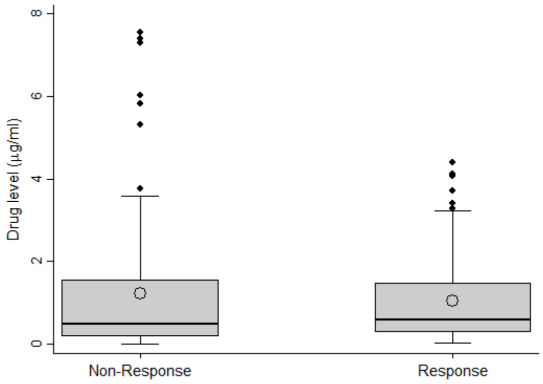

(c) Patients achieving PASI≤1.5 vs PASI>1.5

n = 198 and 82 samples in each group respectively

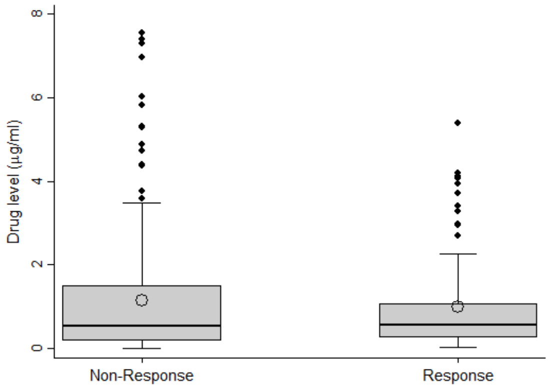

The middle line is the median, grey dots are the means, ends of boxes are the lower and upper quartiles, black dots are outliers (values more than or equal to 1.5 times

© 2019 Tsakok T et al. *JAMA Dermatol*.

the interquartile range from the lower and upper quartiles), whiskers show the minimum and maximum values (unless there are outliers, in which case they are 1.5 times the interquartile range from the lower and upper quartiles).

© 2019 Tsakok T et al. *JAMA Dermatol*.

eFigure 3. Boxplots Comparing Early Drug Levels by 6-Month Response

(a) PASI90 response vs non-response

n = 73 and 46 samples in each group respectively

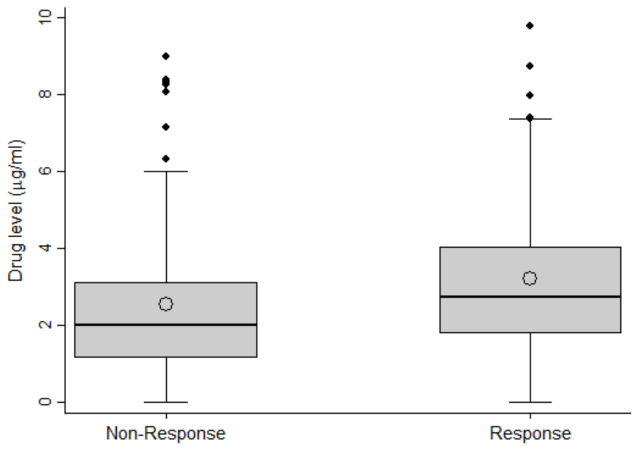

(b) Patients achieving PASI≤1.5 vs PASI>1.5

n = 132 and 59 samples in each group respectively

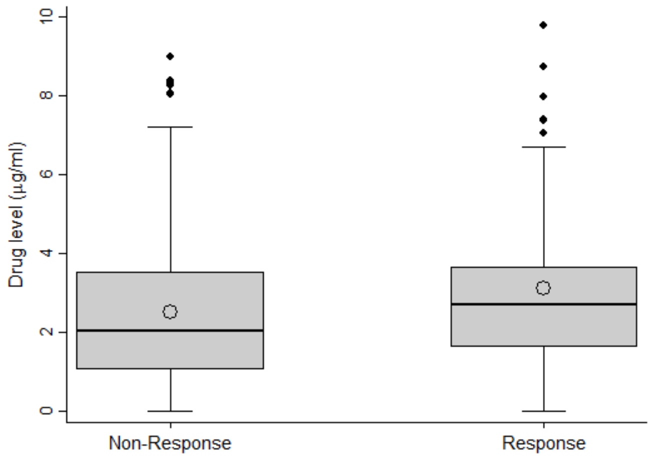

The middle line is the median, grey dots are the means, ends of boxes are the lower and upper quartiles, black dots are outliers (values more than or equal to 1.5 times the interquartile range from the lower and upper quartiles), whiskers show the minimum and maximum values (unless there are outliers, in which case they are 1.5 times the interquartile range from the lower and upper quartiles).

© 2019 Tsakok T et al. *JAMA Dermatol*.

eFigure 4. Boxplots Comparing Early Drug Levels by 6-Month Response, Split by Ustekinumab Dose

(a) PASI90 response vs non-response

n = 38, 30, 35, 16 samples in each group respectively. The red boxes correspond to 45mg and the blue boxes correspond to 90mg.

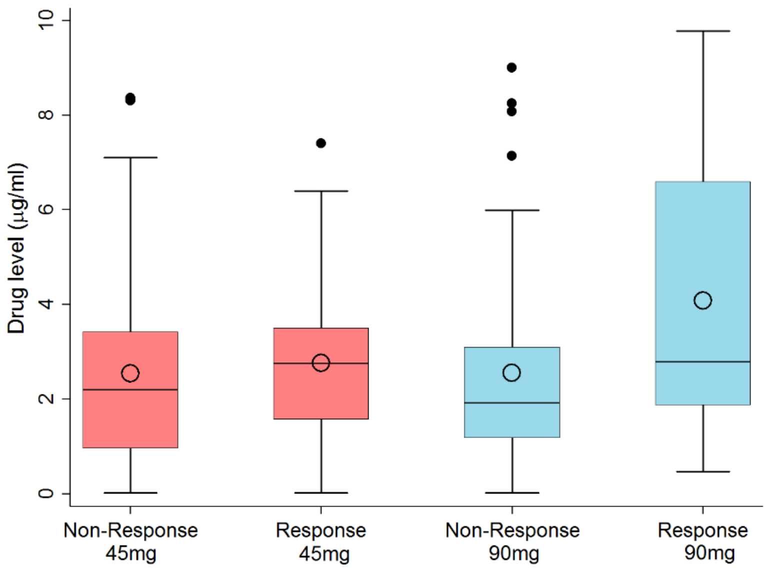

(b) Patients achieving PASI≤1.5 vs PASI>1.5

n = 74, 40, 58, 19 samples in each group respectively. The red boxes correspond to 45mg and the blue boxes correspond to 90mg.

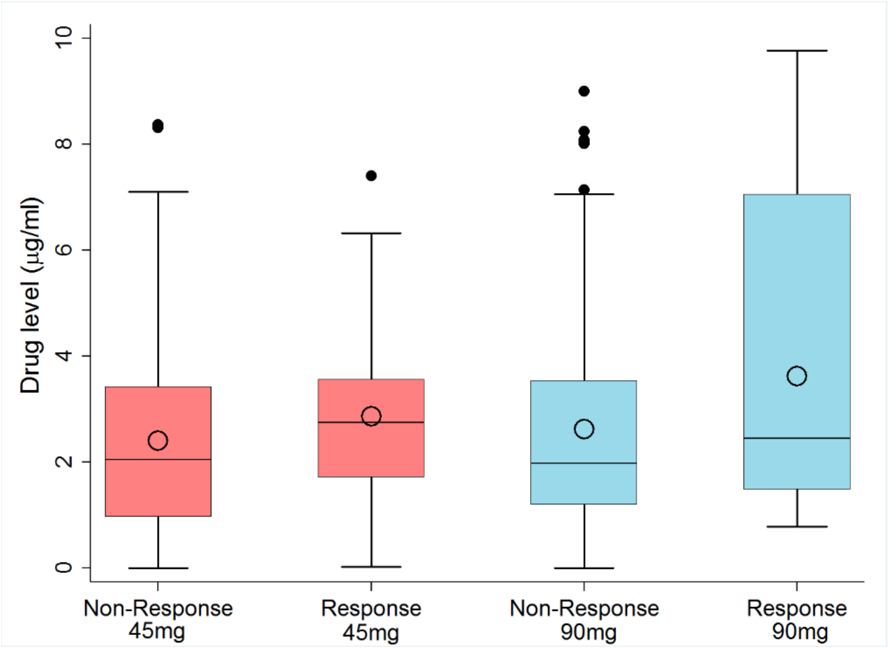

The middle line is the median, red/blue dots are the means, ends of boxes are the lower and upper quartiles, black dots are outliers (values more than or equal to 1.5

© 2019 Tsakok T et al. *JAMA Dermatol*.

times the interquartile range from the lower and upper quartiles), whiskers show the minimum and maximum values (unless there are outliers, in which case they are 1.5 times the interquartile range from the lower and upper quartiles).

© 2019 Tsakok T et al. *JAMA Dermatol*.

eFigure 5. Boxplots Comparing Early Drug Levels by Weight and Ustekinumab Dose

(a) Weight <100 kg vs ≥100 kg

n = 76 and 38 samples in each group respectively

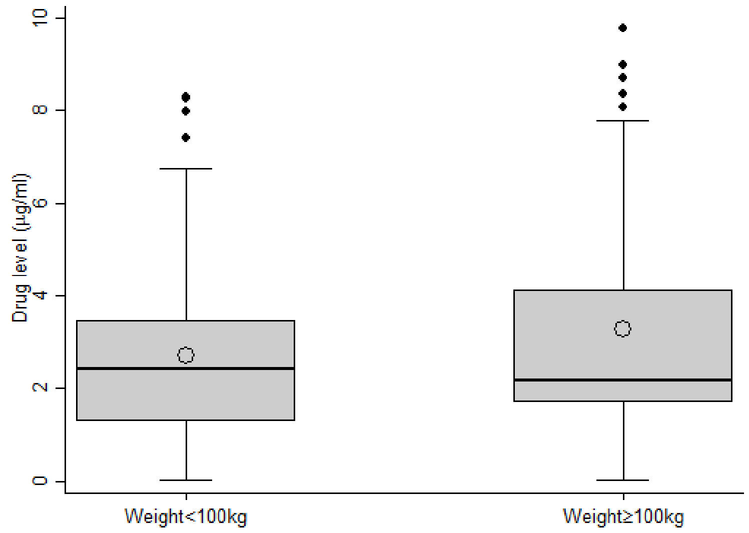

b) Ustekinumab dose 45 mg vs 90 mg

n = 68 and 51 samples in each group respectively

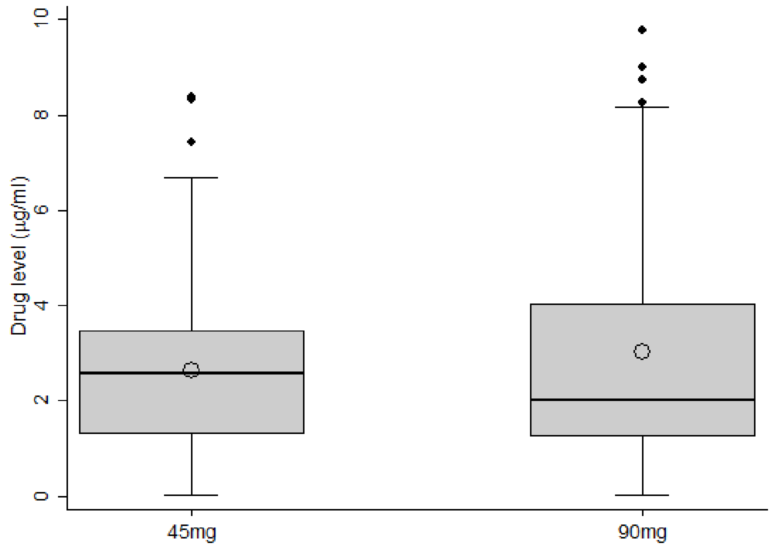

The middle line is the median, grey dots are the means, ends of boxes are the lower and upper quartiles, black dots are outliers (values more than or equal to 1.5 times the interquartile range from the lower and upper quartiles), whiskers show the minimum and maximum values (unless there are outliers, in which case they are 1.5 times the interquartile range from the lower and upper quartiles).

© 2019 Tsakok T et al. *JAMA Dermatol*.
